# Supplementary material for: Are carbon emissions trading and green financial instruments synergistic? -Comprehensive quantitative research based on content analysis
Source: PLoS One. 2024 Mar 7;19(3):e0298601. doi: 10.1371/journal.pone.0298601 (PMC10919601; doi:10.1371/journal.pone.0298601)
Supplement: S1 Data — (ZIP) [file pone.0298601.s001.zip › Data/地级市Z_F工作报告.zip!/║■▒▒/╩«╤▀╩╨/└┤╫╘╣1⁄2╓┌║┼í╛┬φ┐╦╩2╛▌═°í┐_2003.docx]

各位代表：

　　现在，我代表市人民政府向大会作政府工作报告，请予审议，并请各位政协委员和其他列席会议的同志提出意见。

　　一、2002年工作回顾

　　2002年，在市委的正确领导下，全市上下认真贯彻落实“三个代表”重要思想和党的十六大精神，始终把发展作为执政兴市的第一要务，抢抓历史机遇，着力创新环境，大力推进改革，扩大对外开放，加快发展步伐，两个文明建设取得显著成效。

　　（一）国民经济高速增长，人民生活水平稳步提高。

　　我市经济继续高速高效运行。全年实现国内生产总值231.5亿元，同比增长13.9%，完成计划的105.7%；实现财政收入20.86亿元，增长10.1%，完成计划的105.5%，其中地方一般预算收入8.08亿元，增长11.9%。

　　农村经济平稳增长。农业结构调整显现成效。全市大幅调减粮食面积，着力发展多种经营，黄姜产业发展迅速，水果、蔬菜、茶叶、食用菌全面丰收，畜禽和水产品产量稳定增长，弥补了农业受灾、粮油减产的损失，实现农业增加值22.9亿元，增长5.1%。乡镇企业发展步伐加快。全年实现增加值23亿元，增长14.4%。打工经济异军突起。全年实现劳务收入11.13亿元，增长52%。生态环境和基础设施建设成果丰硕。全市治理水土流失面积220平方公里，退耕还林和荒山造林60.1万亩，新建沼气池8400口，解决了15.8万人的饮水困难和71个村通路、15个村通电问题。

　　工业经济实力进一步增强。着力办好“十件实事”，支持服务东风公司实施国际合作，汽车产业增势强劲，全年实现产值335.7亿元，增长33.6%。在以东风公司为龙头的汽车主导产业带动下，全市规模以上工业企业完成增加值112.5亿元，实现利润37.9亿元，分别增长18.1%和221.9%；工业经济效益综合指数161.9，同比高43.1个百分点。

　　新兴产业不断壮大。全年接待游客478.5万人次，实现旅游总收入19.8亿元，分别增长9.4%和13.8%；黄龙滩电厂扩建工程动工兴建，陡岭子电站竣工投产，松树岭电站正式开工建设，全市新增装机容量8.25万千瓦；清华药业被确定为国家中药现代化科技产业基地，全市医药工业实现产值5.3亿元，增长54.9%；武当山珍基地发展到10万多亩，“武珍”牌产品在武汉、北京形成稳定的销售渠道。

　　金融运行平稳。全市金融机构年底各项存款余额270.1亿元，比年初增加42.7亿元；各项贷款余额173.9亿元，比年初增加11.9亿元。

　　人民生活水平稳步提高。全社会消费品零售总额83.5亿元，增长11%。城镇居民人均可支配收入5860元，增长14.9%；农民人均纯收入1714元，增长8%。城市居民最低生活保障工作取得重大突破，农村8万贫困人口解决温饱问题。

　　（二）改革力度进一步加大，对外开放成效明显。

　　各项改革不断深入。农村税费改革取得阶段性成果，全市农民负担总额下降37.6%，耕地亩平负担下降37.2%，调动了农民生产积极性。国有集体企业改革稳步推进，改制后的企业经营状况明显改观。市县乡机构改革工作全面完成，顺利通过了省检查验收。

　　对外开放取得新进展。我市成功举办第五届武当国际旅游节，在上海、北京、厦门、深圳、香港、澳门等地举办或参与大型招商引资活动，取得明显成效。全市新批三资企业17家，投资总额7362万美元；直接利用外资2405万美元，增长59.6%。外贸主体队伍进一步扩大，新增获得进出口经营权企业19家。外贸出口3477万美元，增长28.1%。利用外资和外贸出口超额完成省下达的任务。

　　（三）重点建设步伐加快，经营城市领域扩大。

　　固定资产投资持续增长。全社会完成固定资产投资50.7亿元，增长20.1%。“四个一”工程进展顺利。服务南水北调中线工程的前期工作全面展开，武当山军民合用机场建设进入实施阶段，银武高速公路十漫段前期工作稳步推进，多科性大学组建工作有了明显进展。神定河污水处理一期工程竣工运行,二期工程开工建设;汉十高速公路许土段工程、市体育运动中心、太子坡景区建设、秦岭山区农业综合开发项目、日援汉江防护林工程等进展顺利；汉江铝业三期工程、天神粉状乳化炸药技改项目等正在抓紧推进。

　　城镇建设步伐加快。新修编的《十堰市城市总体规划》通过省政府审批。全市城镇建设投入2.05亿元。城区车城西路、汉江路、朝阳北路、湖南路、广东路、光明小区道路和朝阳中路改造圆满完成；武当山火车站竣工运行，十堰火车站改扩建工程完工启用；郧县沿江大道、郧西县鄂陕边贸市场、竹溪县鄂陕大道、竹山县沿江大堤、房县西门河综合治理、丹江口市均州一路等工程顺利推进，蒋家堰、上津等9个镇被评为全省口子镇建设先进单位。

　　经营城市迈出新的步伐。市直单位通过土地拍卖、产权置换等多种途径，实现直接融资2.9亿元。城区户外广告整治和市直部门房产清理顺利完成，土地资源配置基本实现市场化运作，经营城市由城区向县市扩展。

　　（四）社会事业全面发展，精神文明建设卓有成效。

　　各项事业长足发展。全市“科技三费”同比增长11%，高新技术产值增长44%；白浪开发区经省政府批准，更名为十堰高新技术产业开发区；先锋模具等6家企业被确定为全国制造业信息化示范企业，水稻地膜覆盖技术推广获全国农业科技进步一等奖。全面改善办学条件，积极推进教育改革，大力推行素质教育，义务教育稳步发展；高中招生1.6万人，增加2000人；中职招生8600人，增加2400人。群众文化丰富多彩，文化产业开始起步。成功举办市第二届全民健身运动会，竞技体育继续保持全省前6名，我市选送的运动员在第十四届亚运会上获得1枚金牌。市广播电视中心大楼动工兴建，69个乡镇、410个村实现光纤联网。社区卫生事业迅速发展，城乡医疗市场逐步规范，就医环境进一步优化。计划生育实现省定目标，人口自然增长率为2.38‰。《鄂西北山区国家级生态功能保护区建设规划》开始实施。

　　精神文明建设成效显著。市交通局、市人寿保险公司、太和医院、市审计局被推荐为新一轮国家文明单位，城区顺利通过省级文明城市验收，张湾区、茅箭区获全省示范社区荣誉称号，“十星级农户”和“国道文明走廊”等农村精神文明创建活动蓬勃开展。民兵预备役建设、“人防”和“双拥”工作取得新的成绩。

　　（五）民主法制建设得到加强，社会大局保持稳定。

　　民主法制建设继续推进。各级政府定期向人大报告工作，向政协通报情况，重大决策征求各方面意见，自觉接受人民的监督。市政府办理人大代表议案1件、意见和建议222件，办理政协委员提案292件，办结答复率达100%。接受群众来访，受理群众来信，听取群众意见，化解各种矛盾，密切了政府与人民群众的关系。深入开展民主评议行风和“学查改”活动，认真组织第五次村民委员会换届选举工作，机关作风建设和基层民主建设得到加强。

　　社会环境和谐稳定。全市安置企业分流人员3.31万人，国有企业下岗职工基本生活费和企业离退休人员离退休费发放率均达100%。强化综合治理责任制，认真排查和化解不安定因素，深入开展“严打”整治斗争，全市刑事案件下降14.1%；坚持不懈地开展同“法轮功”等邪教组织的斗争，认真做好“法轮功”人员的教育转化工作，社会治安局势平稳。

　　在全市两个文明建设中，人事、审计、外事、统计、民族宗教、档案、邮政、电信、地震、气象、水文、移民、对台、社科、老龄、残联、地方志等方面的工作都取得了新的成绩。

　　各位代表，过去一年所取得的成绩，凝聚了全市人民的心血、汗水、智慧和力量！在此，我代表市政府，向辛勤工作在全市各条战线上的广大劳动者，向给予政府工作大力支持的人大代表、政协委员和各民主党派、工商联、无党派人士，向为我市发展作出突出贡献的东风公司、汉江集团、东风金狮轮胎公司等中央、省属企业和驻市解放军、武警部队、公安干警，向所有关心支持我市建设的海内外朋友表示由衷的敬意和感谢！

[FS:PAGE]

　　在充分肯定成绩的同时，我们也清醒地看到，当前我市经济社会生活中还存在不少困难和问题。主要是：市县属国有企业改制步伐不快，机制不活，经济运行质量不高，特别是汽配企业跟不上东风公司发展步伐，面临的形势十分严峻；经济发展环境不优，民营企业办事难、负担重的问题仍然较为严重；农业剩余劳动力转移困难，农民增收渠道不多，农村扶贫开发任务艰巨；对外开放意识不强，经济发展外向度低；城镇下岗职工增多，就业和再就业压力加大；少数政府部门及其公务人员作风不实、办事拖拉等问题较为突出，官僚主义、形式主义仍然存在，以权谋私、权钱交易等腐败现象时有发生。对此，我们将采取有力措施，认真加以解决。

　　二、2003年的总体要求和主要任务

　　市委二届三次全会按照党的十六大和省委八届二次全会精神，紧密联系实际，提出了我市全面建设小康社会的奋斗目标。经济建设的主要目标是，“十五”末全市国内生产总值达到320亿元，按可比价格计算年均增长12%；2010年，国内生产总值500亿元，比2000年翻一番；2020年，国内生产总值950亿元，比2000年增长4.5倍。实现这一目标，关键是要开好头，起好步。21世纪头20年是我市加快发展的重要战略机遇期。东风汽车公司国际化合作，南水北调中线工程实施，武当山机场和银武高速公路十漫段建设，国家新阶段扶贫开发，以及改革开放以来市域经济的持续、快速、健康发展奠定了十堰全面建设小康社会的坚实基础等，都为十堰经济、社会的发展提供了难得的机遇和条件。当然，我们也要清醒地看到，我市虽然总体上基本实现小康，但农村经济发展滞后，农民人均收入远低于全省平均水平，城乡差别大，社会经济发展水平仍然较低，全面建设小康社会任重道远。我们一定要牢固树立忧患意识，增强紧迫感和使命感，抢抓机遇，趋利避害，顺势而谋，乘势而上，加快发展步伐。

　　根据新的形势，2003年全市经济社会发展的总体要求是：高举邓小平理论伟大旗帜，全面贯彻“十六大”精神和“三个代表”重要思想，与时俱进，开拓创新，进一步扩大开放，深化改革，加快调整，创新环境，积极推进工业兴市、生态立市，坚持走新型工业化道路，大力发展特色经济、民营经济、外向型经济和县域经济，健全社会保障体系，加大就业和再就业工作力度，确保社会稳定，迈好全面建设小康社会的第一步。

　　按照全面建设小康社会的总体要求，2003年我市经济和社会发展的主要预期目标是：国内生产总值增长9%左右；固定资产投资增长9%；社会消费品零售总额增长9%；财政收入增长8%，其中地方一般预算收入增长2.6%；外贸出口增长10%，利用外资增长25%；农民人平纯收入增长5%，城镇居民人均可支配收入增长6%；城镇登记失业率控制在4.5%以内；人口自然增长率控制在省定目标以内。

　　围绕上述目标，我们要重点抓好十个方面的工作。

　　（一）全面提高对外开放水平。

　　加大招商引资力度。最大限度地发挥市场主体的作用，将企业推向招商引资的最前沿。把以优势资源为特色的生态农业、以汽配产业为龙头的现代工业、以旅游开发和区域性市场建设为代表的第三产业全面推向国际市场，用最优秀的企业、最优良的资产、最有开发价值的资源、最有发展前景的项目与实力雄厚的外商合资重组，引进增量，激活存量。拓宽招商引资领域，把招商引资从经济建设向科技、教育、文化、卫生、体育等领域拓展。不断探索招商引资新途径，通过多种形式和渠道，宣传我市特色项目，密切同外商的联系，增加签约机会。以韩国、日本和台湾、长江三角洲、珠江三角洲为重点，开展招商引资活动。精心策划组织在日本和韩国举办的汽车零部件专题招商会，务求取得实效。

　　积极参与国际市场竞争。紧盯国际市场需求，建设特色出口产品基地，大力发展特色出口商品。积极支持和帮助企业申办进出口经营权，充分利用广交会、高交会等贸易平台，努力扩大外贸出口。开拓国际劳务市场，发展对外劳务合作。

　　完善对外开放措施。积极创造条件，加快对外开放的基础设施建设。提高政府工作部门的服务质量和水平，对外商投资企业和出口大户实行上门服务，现场解决问题。转换政府驻外机构职能，把招商引资作为驻外机构的首要任务。不断完善招商引资和外贸出口责任制，强化责任，奖优罚劣。用足用好出口奖励资金，加大出口退税力度，调动企业扩大出口的积极性。

　　（二）坚定不移地推进市场化改革。

　　加快推进企业改革。认真执行政策，依法规范程序，在落实职工补偿和保障措施的前提下，因企制宜地采取各种方式，全力推进中小企业民营化。积极引导已改制企业，按照现代企业制度规范运作。

　　继续深化农村改革。巩固农村税费改革成果，大力推进配套改革，建立健全农民负担监管体系、涉农负担监控体系和农业税收征管体系，认真落实减人、减事、减支的各项措施，努力实现“三个确保”。按照依法、自愿、有偿的原则，运用市场机制推动农业规模经营和山水资源开发。

　　有序推进国有资产管理体制改革。重点抓好组建市级国有资产管理机构、对国有资产实行分类管理、建设国有资产监管考核体系三项工作。通过对国有资产实行科学规范的经营管理，实现国有资产保值增值。

　　深入推进行政管理体制改革。按照精简、统一、效能的原则，进一步推进政府机构改革，实行政企、政事分开，切实把政府职能转到经济调节、市场监管、社会管理和公共服务上来；深化行政审批制度改革，实现行政审批的法制化和科学化。进一步完善政府工作规程，努力建设“廉洁、勤政、务实、高效”政府。

　　（三）大力发展县域经济。

　　坚持县域经济以民营经济为主体。实行政策扶持。从市场准入、投融资、税收、土地使用、对外贸易等方面进一步完善政策，实行与公有制经济同等待遇；鼓励和支持民营企业采取承包、租赁、并购等形式参与国有企业重组，实行国有民营、民有民营。拓宽发展领域。鼓励民营企业向一、二产业延伸，鼓励民营企业在发展新兴产业中挑大梁，鼓励民营企业参与房地产开发和基础设施建设。优化发展环境。规范收费行为，下重手整治“三乱”，建立和完善信息咨询、技术培训等服务体系。

　　加速农业工业化、产业化进程。立足资源优势，发展农副产品深加工，建设工业园区，探索特色工业化之路。以资源换资金、换技术、换市场，引进一批农产品加工龙头企业；采取政策倾斜、要素重组等措施，努力培育特色农副产品加工的龙头企业。建立龙头企业和农户利益共享、风险分担的运行机制，让农民从产业化经营中获得更多实惠。加强流通中介组织建设，推进生产、加工、销售一体化经营，促进农业产业化健康发展。大力发展“打工经济”，拓宽农民增收渠道。

　　不断提高农村城镇化水平。以加强城镇基础设施建设为突破口，改善企业发展环境；以科学制定城镇体系规划为突破口，合理引导城镇的分工布局；以扩大就业为突破口，引导农民进城务工经商。把小城镇发展与乡镇企业改造升级和农业产业化建设结合起来，以工业化带动城镇化，以城镇化促进农业剩余劳动力转移，形成人口向城镇集中、企业向工业园区集中、土地向经营能手集中的良性互动局面。大力发展边贸流通、风景旅游、资源开发、农副产品加工等各具特色的小城镇。突出抓好县城建设，重点抓好基础较好的中心集镇和“口子镇”建设。坚决消除城镇发展的体制和政策障碍，认真落实和不断完善户籍、土地、投资、子女入学等方面的优惠政策，加速农村城镇化进程。

　　加大新阶段扶贫开发力度。组织实施新阶段扶贫开发规划，切实推进283个重点村村级规划的实施，总体解决8万贫困人口的温饱问题。积极争取中央、省加大对我市新阶段扶贫开发的投入，组织好部门、单位对口扶持，促进革命老区、贫困地区农民尽快脱贫致富。加强农业基础设施建设，大力改造低产田，新建各类水利工程1.2万处，解决16万人饮水困难。

[FS:PAGE]

　　（四）积极推进工业兴市。

　　全力支持服务“新东风”的发展。继续做好政企共建工作，加强市政基础设施建设，集中整治东风公司周边环境，深入开展“打假保东风”活动，维护良好的生产生活秩序。积极争取政策，创新环境，大力支持东风公司改革发展。

　　加快汽配工业的调整重组步伐。大力调整产品结构，支持车身、内装饰、座椅等系列产品和燃油箱、消声器、车桥、车架等部分总成，进入东风有限公司采购体系，扩大配套份额。积极推进企业联合重组，利用我市汽车零部件工业的地域优势、成本优势、市场优势，与日本、韩国及台湾、江浙等地零部件供应商开展多种形式的合作；支持技术先进、实力较强的汽配企业与东风公司专业厂合资合作，争取成为东风公司的核心企业；支持东风金狮轮胎公司与马来西亚银石轮胎公司合作；支持汉江集团等中央、省属企业的改革与发展；支持优势企业兼并、收购劣势企业，实现低成本扩张。通过调整重组，使我市汽车零部件工业从小而全向专业化、规模化方向发展，从分散经营向集团化方向发展，从单一产品结构向模块化方向发展。

　　培育壮大新兴产业。大力发展水电产业，确保孙家滩、瓦房坪、顺水坪等电站建成投产，加快松树岭电站、黄龙滩扩建工程的建设步伐，力争鄂坪、柳园铺等电站开工兴建，抓好孤山、三里坪、夹河关等电站的前期工作，推进“全国水电电气化县”建设。发展壮大医药产业，加强中药材GAP基地建设，支持清华药业、麦克药业、百科药业、康迪制药、安泰激素、丹澳医化等企业做大做强。突破性发展绿色食品产业，加强绿色食品基地建设，注重创立精品名牌，提高我市绿色产品的市场占有率。

　　加强企业家队伍建设。积极引导机关干部、科研人员、大中专毕业生到企业工作，领办和创办企业，着力提高他们驾驭市场、参与竞争尤其是参与国际竞争的能力。制定政策，创新机制，为企业家脱颖而出创造良好的创业环境和社会氛围。建立健全企业家选拔任用和奖惩激励机制，培育和造就一支职业化、高素质的企业家队伍。

　　（五）加速发展第三产业。

　　大力发展旅游产业。加大区域旅游资源和旅游产品的开发、整合力度，努力形成以武当山为龙头，以十堰城区为中心，以县市和周边著名景区为网点，由精品线路组成的大旅游格局。总结推广张湾区月亮湖山庄、郧县金沙湾、丹江口市九龙岛等地的经验，通过出让经营权等方式筹措资金，加快景区、景点开发，着力推进武当山太子坡生态旅游等项目建设。强化措施，依法保护文化遗产安全，抓紧做好准备工作，确保武当山古建筑群通过世界文化遗产复核。加大宣传促销力度，进一步拓展国内外旅游市场，力争接待游客人次、旅游总收入分别增长10%以上。

　　建立健全市场体系。加快武当商城、堰中蔬菜批发市场二期工程、朝阳路小副食品交易市场三期工程、肉禽蛋水产交易市场建设，规范发展旧车交易市场，建立服务城乡、辐射周边的特色产品集散地。以汽配城为载体，不断扩大汽车及其配件产品的市场规模，努力把汽配城发展成国内最大的汽车零部件集散中心。全面放开住房二级市场，把房地产业培育成我市新的经济增长点。积极发展人才、劳务、技术、资金和产权交易等要素市场，创造各类市场主体平等使用生产要素的环境。

　　不断创新流通方式。积极推进大型商贸流通企业走强强联合之路，壮大实力，提高效益。总结推广京华超市、丰融超市、寿康永乐公司等流通企业的发展经验，充分发挥新业态的优势，大力推行代理制、委托制，推进以连锁经营、物流配送和电子商务为主的流通现代化建设。

　　大力发展服务业。在稳定发展传统服务业的同时，鼓励各种经济成分投资新兴服务业。积极发展代理、咨询、评估、拍卖、仲裁等中介组织，加强行业协会和商会建设。加快发展社区医疗、文化、家政、物业管理等社区服务业，推进社区服务向网络化、产业化、社会化方向发展。

　　（六）全力抓好重点项目建设。

　　加快在建项目建设。全线贯通北京路，做好沿线土地开发工作。尽快完成郧县、丹江口市农网和城区电网改造。确保市体育中心在第十一届省运会之前竣工。加速推进汉十高速公路许土段、丹江二桥、市污水处理厂二期工程、热电厂二期工程等项目建设。重点抓好十通高速公路运输车、“戎马”汽车差速传动装置、三相感应式同步电机、东风金狮全钢子午胎和高档轿车轮胎等技改项目。

　　开工一批新建项目。全面推进“四个一”工程建设。加快武当山机场改造步伐；继续做好银武高速公路十漫段施工前的准备工作，争取年内动工；切实做好丹江口水利枢纽加坝前的各项工作；抓紧做好多学科大学校园整体规划，多渠道筹措资金，推进基本建设。切实搞好干线路网改造和通乡油路建设。认真组织实施丹江口、黄龙滩库区后期扶持规划。力争丹江城区汉江公路大桥动工兴建。大力支持丹江铝业10万吨铝扩能改造工程建设。积极支持配合东风公司争取国债项目，与日产合资开发新车型。

　　筹划一批储备项目。按照基础性项目与生产性项目并重并举的原则，筹划一批符合国家投资方向和产业政策的重大项目。重点围绕南水北调中线工程水源区保护，争取流域治理、污水处理、生态建设和文物保护项目；围绕库区移民，争取移民安置、实物补偿和可持续发展项目；围绕新阶段扶贫开发，进一步争取扶贫项目，做好生态移民项目的衔接工作。注重项目质量，扩大项目数量，加大项目争取力度，开创项目工作新局面。

　　推进经营城市工作。规范和完善土地市场，采用招标、拍卖、挂牌出让等方式集中经营城市土地。盘活行政事业单位房产资源，提高国有房产使用效益。推进市政公用行业市场化进程，鼓励民间资本、外来资本采取独资、合资、合作等形式，参与基础设施建设。加强广告管理，对广告经营权实行公开招标拍卖。加快十堰区域性中心城市建设步伐，提升城市功能，壮大经济实力，发挥辐射带动作用。

　　（七）做好财税金融工作。

　　继续推进财政改革，努力增收节支，确保财政收支基本平衡。综合运用财税政策和财政手段，支持基础财源、骨干财源和新兴财源建设。坚持依法治税，对重点地区、重点税源实施有效监控，确保主体税种稳定增长，挖掘增收潜力，增加地方可用财力。按照公共财政体制的要求，深化财政改革，积极推进部门预算，完善国库集中支付、政府集中采购制度；严格实行“收支两条线”，加强和改进预算外资金管理。优先保证工资发放、社保支出、再就业投入、机关正常运转和法定支出，从严控制一般性支出，下大力降低行政运行成本。

　　改进金融服务，支持经济发展。金融部门要进一步调整信贷结构，积极向上争取信贷规模和指标，加大对优势产业、骨干企业和重点项目的信贷支持力度。以担保中心为支点，加强对中小企业和民营企业的信贷投入。管好用好支农再贷款，继续推广农村小额信贷，支持农业和农村经济发展。进一步抓好信用环境的专项治理，全面创建金融安全区，强化金融监管，打击恶意逃废银行债务的行为，有效防范和化解金融风险。

　　（八）大力发展社会事业。

　　加大科教兴市力度。加快十堰高新技术开发区建设步伐，积极申报国家火炬计划汽车零部件产业基地。继续扩大对外科技合作，支持企事业单位与国内外知名企业、科研机构和大专院校建立战略协作关系，支持省级重点实验室建设。创建风险投资机制，形成以企业为主体的多元化科技投资体系。用信息技术改造和提升传统产业，着力推进制造业信息化示范工程。大力扶持民营科技型企业发展，促进民营经济上档次、上水平。加强农业标准化和农产品质量检验监测体系建设，推广普及地膜覆盖、节水灌溉等先进适用技术，提高农业科技含量；推广使用农业机械，提高农业劳动效率。巩固“普九”成果，完善农村义务教育以县为主的管理体制，扩大高中招生规模，全面推进素质教育。注重解决税费改革后农村义务教育中出现的新问题，采取社会救助、助学贷款等方式，帮助贫困学生完成学业。整合教育资源，大力发展职业教育、成人教育和继续教育，支持社会力量办学，构建终身教育体系。高度重视人才的培养、引进和使用，完善优惠政策，逐步改善各类人才的工作、生活条件。

[FS:PAGE]

　　发展文体、卫生和计划生育事业。发掘汽车文化、武当文化、汉水文化资源，发展文化产业；逐步完善城乡文化基础设施，广泛开展丰富多彩的群众文化活动；坚持“扫黄打非”、打击盗版侵权行为，净化文化市场。发展广播电视事业，将广播电视光纤网延伸到所有乡镇和大部分行政村。承办好第十一届省运会，力争我市团体总分或金牌数进入前6名。整合医疗资源，发展卫生产业；加强卫生检疫和安全防范，提高疾病控制和卫生执法水平；实施“农村卫生网络建设工程”和“社区卫生服务进万家工程”，满足城乡居民的健康需求。全面贯彻《人口与计划生育法》，坚决执行“一票否决制”，稳定低生育水平，努力提高出生人口质量。

　　坚持生态立市。我市是南水北调中线工程水源区和国家生态功能区，必须坚持生态立市与工业兴市并重。进一步加大水源区水质污染的治理力度，高度重视并切实解决黄姜加工企业的污染问题，确保调水水质。加快城市污水处理系统建设，逐步扩大城市集中供热范围，改善大气环境质量，积极创建国家环保模范城市。继续加强生态项目建设，确保完成62万亩退耕还林和荒山造林任务，推进生态家园建设，兴建农村沼气池1.5万口，治理水土流失面积280平方公里。

　　加强精神文明建设。全面实施《公民道德建设纲要》，让广大公民形成良好的社会公德、职业道德和家庭美德。以创建国家级文明城市为目标，积极开展文明社区、文明单位、文明农户等系列创建活动。积极支持部队和国防建设，深入开展国防教育，继续搞好“双拥”活动，确保全国、全省“双拥”模范城四连冠。

　　（九）着力优化发展环境。

　　坚持依法治市，营造规范严明的法制环境。加强民主法制建设。继续开展“四五”普法教育，重点提高各级领导干部、司法人员和行政执法人员、青少年学生、企业经营管理人员的法律素质。各级政府要严格按照法定的权限和程序履行职责，认真执行人大的各项决议，自觉接受人大监督和政协民主监督，认真听取各民主党派、工商联、无党派人士和人民团体的意见，充分发挥各类专家和决策咨询机构的作用，推进决策的民主化和科学化。坚持依法行政。深入推行执法责任制和执法违法责任追究制，强化行政执法监督。成立综合执法局，相对集中行政处罚权，促进公正执法、文明执法。

　　整顿和规范市场秩序，营造诚实守信的信用环境。继续严厉打击制售假冒伪劣商品的违法犯罪活动，加强汽车和配件市场的监管，重点整顿规范医疗器械和药品购销市场、建筑市场、集贸市场，继续抓好“放心肉”、“放心菜”工程。着力打造“信用十堰”，逐步建立企业和个人信用档案网络，健全信用体系，建设诚信社会。

　　做好社会稳定工作，营造安定和谐的社会环境。千方百计做好就业和再就业工作，坚持以发展促进就业，全面落实各项促进就业政策，大力发展就业容量大和市场需求旺的劳动密集型产业、社区服务业和民营企业，拓宽就业渠道；加强劳动力市场建设，做好中介服务和就业培训工作；加快建立再就业补助基金，为特困下岗职工提供再就业援助；完善基层劳动保障工作体系，大力开发公益性就业岗位，力争今年净增就业岗位2万个，安置就业2.5万人。切实加强社会保障制度建设，继续抓好“两个确保”和“三条保障线”的衔接工作；加大社会保险扩面征缴力度，多渠道筹集和积累社会保障基金；建立社会捐赠网络和社会救助保障体系，切实关心困难企业职工、受灾群众、特困家庭和优抚对象的生产生活。加强社会治安综合治理，认真排查并着力解决影响社会安定的突出问题，严密防范和依法严厉打击各种犯罪活动及“法轮功”等邪教组织。强化安全生产责任，坚决消除各种安全隐患，确保人民群众生命财产安全。

　　（十）加强政府自身建设。

　　进一步加强学习。面对全面建设小康社会的繁重任务，各级政府及其工作人员特别要把学习“三个代表”重要思想摆在首位，抓好知识更新和业务培训，帮助广大干部掌握更多的新知识、新技能和新本领。要适应建设学习型社会的要求，确立终身教育的观念和全员培训的目标，着力提高科学判断形势、驾驭市场经济、应对复杂局面、依法行政和总揽全局的能力，大力提高干部队伍素质。

　　转变工作作风。强化公仆意识，恪守为民之责，多办利民之事。坚决反对高高在上、脱离群众的官僚主义和只图虚名、不求实效的形式主义，坚决查处弄虚作假、欺上瞒下、强迫命令、滥用民力的恶劣行为，坚决刹住挥霍公款、贪图享受的奢侈浪费之风。大力精减会议、文件，减少应酬，深入实际，深入群众，了解民意，关心群众疾苦，把群众的冷暖时刻放在心上，及时解决群众关心的热点难点问题，积极主动地为基层服务、为群众排忧解难。继续深入开展行风评议活动，推行政务公开。完善行政服务中心的职能，提高服务质量。强化政府责任体系，明确责任，赏罚分明。

　　深入开展反腐败斗争。全面落实廉政建设责任制，对政府机关工作人员严格要求、严格教育、严格管理、严格监督。坚持以“制权、管钱、用人”为主线，加强对权力运行的约束，努力从体制上、机制上铲除腐败滋生和蔓延的土壤。继续抓好领导干部经济责任审计，切实解决“政府权力部门化、部门权力利益化”的问题，重点查办“三机关一部门”和金融、房地产、土地招标拍卖、建筑工程等领域的案件，真正做到旗帜鲜明地弘扬正气，理直气壮地狠煞歪风，毫不手软地惩治腐败，以廉政建设的实际行动取信于民，以良好的政风促进整个社会风气的好转。

　　各位代表，做好2003年的工作，对于我市全面建设小康社会，意义十分重大。让我们高举邓小平理论伟大旗帜，努力实践“三个代表”重要思想，全面贯彻党的十六大精神，在市委的正确领导下，与时俱进，扎实工作，为实现全面建设小康社会的宏伟目标而努力奋斗！
